# A survey of clinicians on the use of artificial intelligence in ophthalmology, dermatology, radiology and radiation oncology

**DOI:** 10.1038/s41598-021-84698-5

**Published:** 2021-03-04

**Authors:** Jane Scheetz, Philip Rothschild, Myra McGuinness, Xavier Hadoux, H. Peter Soyer, Monika Janda, James J.J. Condon, Luke Oakden-Rayner, Lyle J. Palmer, Stuart Keel, Peter van Wijngaarden

**Affiliations:** 1 Level 7, Centre for Eye Research Australia, Royal Victorian Eye and Ear Hospital, 32 Gisborne Street, Melbourne, VIC 3002 Australia; 2 Ophthalmology, Department of Surgery, University of Melbourne, Melbourne, Australia; 3 Centre for Epidemiology and Biostatistics, Melbourne School of Population and Global Health, University of Melbourne, Melbourne, Australia; 4 Dermatology Research Centre, The University of Queensland Diamantina Institute, The University of Queensland, Brisbane, QLD Australia; 5 Dermatology Department, Princess Alexandra Hospital, Brisbane, QLD Australia; 6 Centre for Health Services Research, The University of Queensland, Brisbane, Australia; 7 School of Public Health, University of Adelaide, Adelaide, Australia; 8 Australian Institute for Machine Learning, University of Adelaide, Adelaide, Australia

**Keywords:** Health policy, Software, Health care, Health occupations, Medical research, Mathematics and computing

## Abstract

Artificial intelligence technology has advanced rapidly in recent years and has the potential to improve healthcare outcomes. However, technology uptake will be largely driven by clinicians, and there is a paucity of data regarding the attitude that clinicians have to this new technology. In June–August 2019 we conducted an online survey of fellows and trainees of three specialty colleges (ophthalmology, radiology/radiation oncology, dermatology) in Australia and New Zealand on artificial intelligence. There were 632 complete responses (n = 305, 230, and 97, respectively), equating to a response rate of 20.4%, 5.1%, and 13.2% for the above colleges, respectively. The majority (n = 449, 71.0%) believed artificial intelligence would improve their field of medicine, and that medical workforce needs would be impacted by the technology within the next decade (n = 542, 85.8%). Improved disease screening and streamlining of monotonous tasks were identified as key benefits of artificial intelligence. The divestment of healthcare to technology companies and medical liability implications were the greatest concerns. Education was identified as a priority to prepare clinicians for the implementation of artificial intelligence in healthcare. This survey highlights parallels between the perceptions of different clinician groups in Australia and New Zealand about artificial intelligence in medicine. Artificial intelligence was recognized as valuable technology that will have wide-ranging impacts on healthcare.

## Introduction

Nomograms and clinical algorithms have long played a role in supporting clinical decision-making in medicine. More recently, major advances in research on artificial intelligence (AI) have been applied to medical image analysis with promising results^1,2^. This technology is now poised for clinical application. In order for AI to fully deliver its potential benefits for healthcare, medical practitioners need to understand and embrace the technology. Equally, patients need to entrust aspects of their healthcare to AI systems.

Numerous AI image analysis algorithms have been shown to achieve high level performance, in some instances comparable to human experts, in the detection of a range of diseases in ophthalmology^3–5^, radiology^6–8^, dermatology^9,10^, and in other fields of medicine. A significant milestone in the clinical application of AI came in 2018 with the US Food and Drug Administration approval of the IDx-DR system for the autonomous detection of referable diabetic retinopathy—the first such approval in any field of medicine^2^. Although the application of this technology is nascent, it has the potential to transform aspects of healthcare^11^.

As is true for any new medical technology, the extent to which AI algorithms for screening, diagnosis or prognosis are adopted in medicine will be dependent upon the attitudes of clinicians and patients. Consideration of these attitudes and knowledge gaps will therefore be essential for health systems, medical educators, professional bodies, AI developers and regulators. Few studies have examined clinician perceptions of new AI technologies on healthcare provision and the clinical workforce. Those that have been conducted have surveyed the potential impacts of AI in samples of medical students^12–14^, radiology trainees^15^, radiologists^16–18^, pathologists^19^, psychiatrists^20^, general practitioners (GP)^21^, as well as general physicians, surgeons, and trainees^22,23^, with contrasting results. Most surveys indicate that clinicians believe that AI will have a positive impact on their profession^13,16–19,22^. Psychiatrists and general practitioners indicated that AI would not affect their profession^20,21^. In contrast, surveys of medical students^12^ and radiology trainees^15^ have highlighted concerns about the implications of AI for training and employment prospects^24–27^.

Areas of medicine that are most reliant on imaging will be amongst the first to be impacted by advances in AI technologies^28^. These include ophthalmology, radiology/radiation oncology and dermatology. In this study we conducted a survey of fellows and trainees of these specialty colleges in Australia and New Zealand in order to ascertain their current use, understanding and perceptions of AI.

## Results

### Demographics

A total of 632 trainees and fellows from three specialty colleges (305 ophthalmology, 230 radiology/radiation oncology, and 97 dermatology) completed the survey (Supplementary Tables 1 and 2). Completed surveys were received from 20.4% of members of the Royal Australian and New Zealand College of Ophthalmologists (RANZCO) (1279 fellows, 212 trainees), 5.1% of members of the Royal Australian and New Zealand College of Radiologists (RANZCR) (4505 fellows and trainees) and 13.2% of members of the Australasian College of Dermatologists (ACD) (621 fellows, 113 trainees). RANZCR members included both radiologists (n = 199; 86.5% of RANZCR respondents) and radiation oncologists (n = 31; 13.5% of RANZCR respondents). ACD respondents were from Australia, whereas RANZCO and RANZCR respondents were from Australia and New Zealand. Incomplete surveys (n = 85/717, 11.9%) were excluded from analyses (Supplementary Table 3). Responses from completed surveys are summarised in Supplementary Table 4.

Respondents predominantly practiced in metropolitan areas (n = 460, 72.8%), followed by a combination of metropolitan and rural practice (n = 102, 16.1%) and rural practice only (n = 70, 11.1%). Ophthalmologists were more likely to spend at least part of their time in a rural setting than radiologists/radiation oncologists or dermatologists (32.5%, 21.7% and, 23.7% respectively, *p* = 0.064). Respondents from each group had similar years of professional experience (*p* = 0.608) and almost half of all respondents had been in practice for 20 years or more (n = 303, 47.9%) (see Supplementary Table 2).

### Current knowledge and use of artificial intelligence in clinical practice

**Figure 1:**
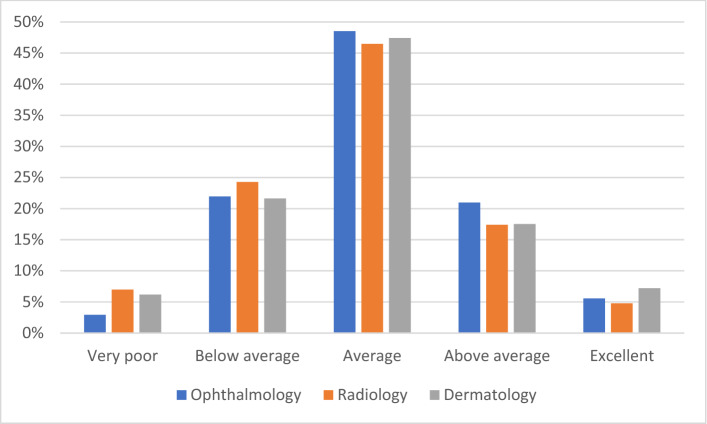
Self-reported knowledge of artificial intelligence and its application in the respondent’s specialty, relative to peers in that field.

**Figure 2:**
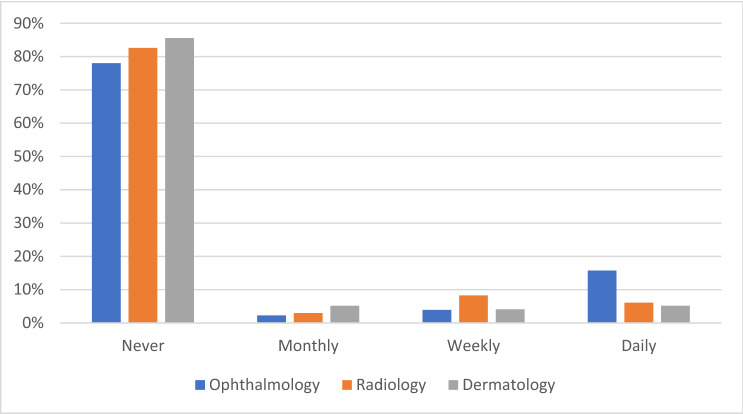
Current frequency of artificial intelligence use in clinical practice.

Almost half of respondents (47.6%; n = 301) rated their knowledge of AI as average relative to their peers, with few rating their knowledge as excellent (n = 35, 5.5%) or very poor (n = 31, 4.9%) (Fig. 1; Supplementary Table 4). Responses were similar across the three professional groups (*p* = 0.542). Most respondents indicated that they had never used AI applications in their work as a clinician (511, 80.9%). Ophthalmologists were more than twice as likely to use AI in their daily clinical practice than radiologists/radiation oncologists or dermatologists (15.7%, 6.1% and 5.2%, respectively, *p* = 0.001) (Fig. 2). Among the 67 ophthalmologists who specified how AI was utilized in their clinics, applications included those for glaucoma progression analysis, optical coherence tomography assessment, diabetic retinopathy image assessment and intraocular lens (IOL) power calculation (Supplementary Table 5). Among the 40 radiologists/radiation oncologists who provided details as to how they use AI, applications included those for automated organ and lesion detection using computer aided detection of computed tomography and magnetic resonance imaging (Supplementary Table 5). Among 8 dermatologists, the most common applications included those for skin lesion surveillance and voice transcription (Supplementary Table 5). Respondents who reported using AI had higher self-reported knowledge of AI than those that did not (*p* = 0.008).

### Perceived impact of artificial intelligence on the profession

**Figure 3:**
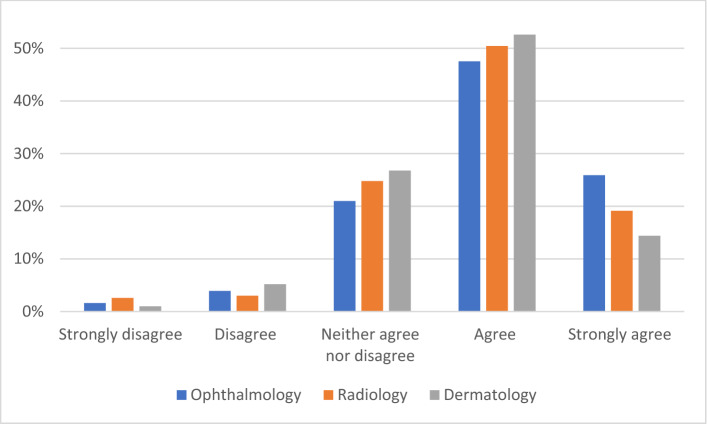
Levels of agreement with the statement “the field of [your specialty] will improve with the introduction of artificial intelligence”.

**Figure 4:**
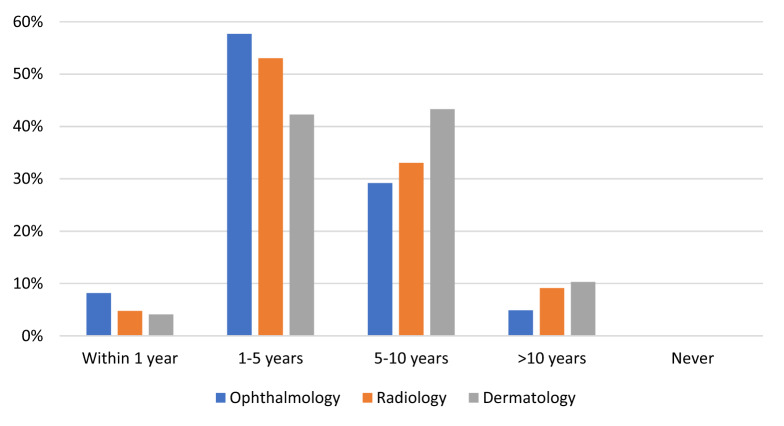
Perceived length of time before artificial intelligence has a noticeable impact on [your specialty].

Most respondents predicted that the introduction of AI would improve their field of practice (n = 449, 71.0% agreed or strongly agreed, Fig. 3). Almost two thirds (n = 379, 60.0%) of respondents believed that it would be ≤ 5 years before AI had a noticeable impact on their specialty (Fig. 4). No respondents reported that AI would never have a noticeable impact on their profession. Respondents who reported using AI predicted that the impact would be apparent sooner that respondents who did not (*p* < 0.001).

**Figure 5:**
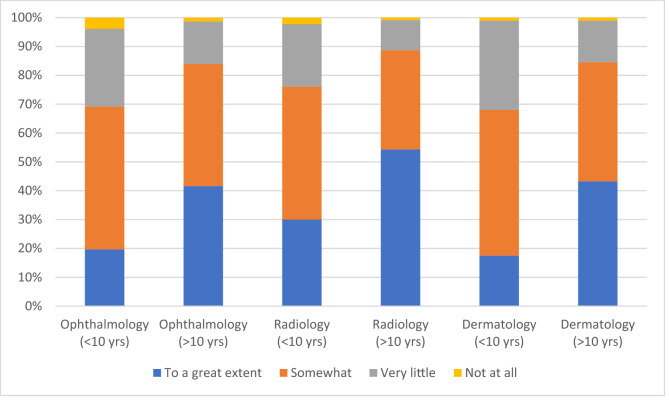
Estimated impact of artificial intelligence on workforce needs within (< 10 years) and beyond (> 10 years) the next decade for each specialty group.

The majority of respondents (n = 452, 71.1%) reported that AI will impact workforce needs ‘somewhat’ or ‘to a great extent’ in these specialty areas of medicine within the next decade. Most (n = 542, 85.8%) respondents believed that AI will impact workforce needs ‘somewhat’ or ‘to a great extent’ beyond the next decade. Radiologists/radiation oncologists were more likely to report workforce needs will be impacted ‘to a great extent’ within the next decade than ophthalmologists or dermatologists (54.4%, 41.6%, and 43.3%, respectively, *p* = 0.04) (Supplementary Table 4; Fig. 5).

The majority of radiologists/radiation oncologists (n = 202, 87.8%) and dermatologists (n = 53, 54.6%) considered that their medical specialist workforce would be impacted by AI to a greater extent than other health professionals in their field of practice (such as radiographers or general practitioners). In contrast, ophthalmologists considered that the impact of AI on the workforce would be greater for optometrists, than for general practitioners or ophthalmologists. Respondents who reported using AI were more likely to report that the introduction of AI would lead to the need for an increase in workforce numbers than did those who did not use AI (*p* = 0.006).

### Acceptable artificial intelligence performance standards and clinical workflows

**Figure 6:**
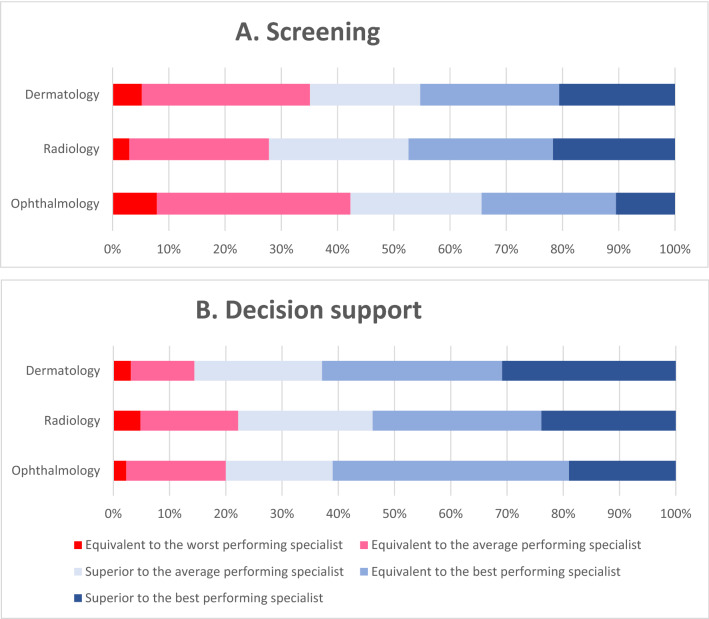
100% stacked bars showing the acceptable level of error for an AI tool used for (**A**) disease screening and (**B**) clinical decision support.

Most survey respondents considered that AI systems would need to achieve performance that was superior to the average performing specialist when applied to screening for disease (n = 405, 64.1%, Fig. 6A) or for diagnostic decision support (n = 506, 80.1%, Fig. 6B).

Radiologists/radiation oncologists and dermatologists were twice as likely as ophthalmologists to consider that AI screening systems should have error levels superior to the best performing specialist (21.7%, 20.6%, and 10.5% respectively, *p* = 0.005, Fig. 6A).

Expectations for system performance were even higher when used for diagnostic decision support by specialists. Accordingly, radiologists/radiation oncologists and dermatologists were each more likely than ophthalmologists to expect AI systems to be superior to the best performing specialist when used for decision support (30.9% and 23.9% and 19.0% respectively, *p* = 0.035, Fig. 6B).

A hypothetical clinical workflow was proposed as follows: “patient clinical images undergo artificial intelligence analysis. A specialist subsequently reviews both the image and the artificial intelligence findings”. Most respondents would consider using this clinical workflow, however significantly more ophthalmologists and radiologists/radiation oncologists were willing to consider this than dermatologists (82.0%, 82.6%, and 67.0% respectively, *p* < 0.001).

### Perceived advantages of the use of artificial intelligence

**Figure 7:**
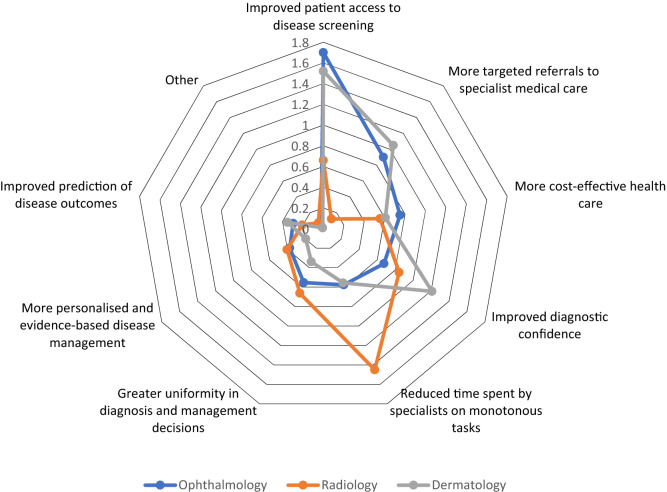
Radar plot showing the highest scoring responses for the greatest perceived advantages of the use of artificial intelligence. Responses were selected from a list of set choices. Plot axes represent the average ranks for all respondents, with higher scores indicating a higher ranking/stronger preference.

The top three ranked potential advantages of AI were (1) improved patient access to disease screening, (2) improved diagnostic confidence, and (3) reduced time spent by specialists on monotonous tasks. The top ranked advantage for ophthalmologists and dermatologists was ‘improved patient access to disease screening’ and the top ranked advantage for radiologists/radiation oncologists was ‘reduced time spent on monotonous tasks’ (Fig. 7).

### Concerns about the use of artificial intelligence

**Figure 8:**
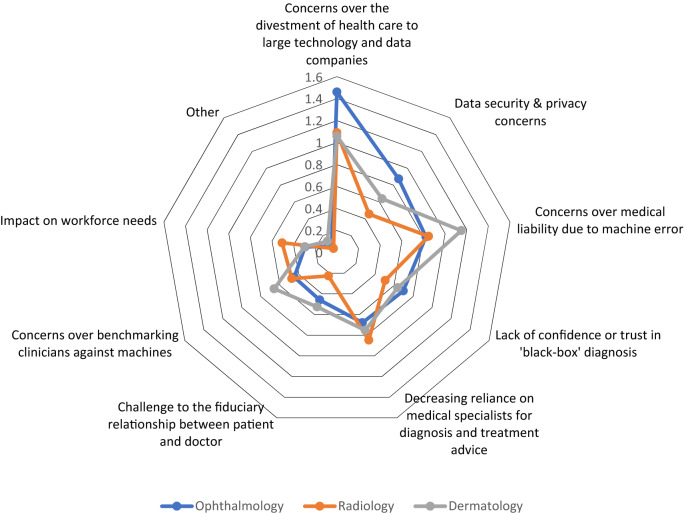
Radar plot showing the highest scoring responses for the perceived concerns or drawbacks of the use of artificial intelligence. Responses were selected from a list of set choices. Plot axes represent the average ranks for all respondents, with higher scores indicating a higher ranking/stronger preference.

The top three potential concerns about the use of AI were (1) concerns over the divestment of healthcare to large technology and data companies, (2) concerns over medical liability due to machine error, and (3) decreasing reliance on medical specialists for diagnosis and treatment advice. The top ranked concern for ophthalmologists and radiologists/radiation oncologists was ‘concerns over the divestment of healthcare to large technology and data companies.’ The top ranked concern for dermatologists was ‘concerns over medical liability due to machine error’ (Fig. 8).

### Preparedness for the introduction of artificial intelligence in clinical practice

A minority of respondents (n = 87; 13.8%) felt that the specialist training colleges were adequately prepared for the introduction of AI into clinical practice. Qualitative analysis of a total of 632 open-ended responses was performed, with similar responses grouped according to theme. Examples of verbatim responses are provided for each of the top five themes in Supplementary Table 6. Many respondents (n = 285) highlighted a need for improved training and education of college members about AI via scientific meetings, seminars, and the training curriculum*.* Some respondents highlighted the need for the colleges to be proactive in their approach in order to safeguard member interests (n = 99), with one participant requesting the college “make sure any changes are in the best interests of patients and [the] profession”. Others endorsed the development of frameworks and/or guidelines for AI implementation by the colleges (n = 30). The formation of sub-committees and working groups with expertise in AI to develop position statements (n = 46) was seen by some as a priority. Other response themes related to colleges taking leadership in relation to AI policy development and licensing (n = 74) and to be intimately involved in the trial and application of emerging AI systems (n = 69). Others called for the development of measures to ensure patient safety (n = 16) and the optimisation of clinical outcomes (n = 17).

Ensuring that patient care remains in the realm of (the specialist) (n = 39) was recognized as a priority. In addition, the implementation of measures to assist clinicians in adapting to changes in care delivery (n = 17) was reported as a goal, with one respondent stating that this will “assist trainees to prepare for the AI era”. Some respondents highlighted a need for colleges to support more AI research (n = 69) and engage with industry, regulators and Government (n = 74). A need for clarity regarding the responsibilities of clinicians who will use the technology was also emphasised. Several respondents highlighted the need for a redefinition of the professional scope of specialists and related healthcare provider groups as AI is deployed more widely in clinical practice.

### Analysis according demographic subgroups

No statistical differences were found for responses based on location of practice (metropolitan, rural or both, *p* > 0.149, Supplementary Table 7). In contrast, differences were noted based on years of clinical experience (Supplementary Table 8). Respondents currently in training were more than twice as likely than qualified specialists to consider that AI will impact workforce needs ‘to a great extent’ within the next decade (*p* < 0.001), despite no differences in self-reported knowledge of AI and its applications between these groups. Similarly, those still in training (n = 44, 66.7%) or with less than 5 years of experience as a specialist (n = 41, 66.1%) were more likely to believe that AI would impact practice ‘to a great extent’ beyond the next decade than were those with 20–30 years of experience or more than 30 years of experience (*p* < 0.001). Those with more than 30 years of experience were less likely to believe that workforce needs would decrease compared to those with fewer years of practice (*p* = 0.007).

### Comparison of responses between radiologists and radiation oncologists

Despite the distinct clinical roles of radiologists and radiation oncologists, no meaningful differences were detected in responses between the two professional groups (*p* ≥ 0.14), with the exception of knowledge of AI (radiation oncologists were more likely to rate their knowledge as below average compared to their peers, *p* = 0.02) and the number of years before AI has a noticeable effect on their field (radiation oncologists were more likely to believe that the effect will be noticeable within the next five years, *p* = 0.01). It is possible that responses from a larger sample of specialists in these fields may identify group differences.

## Discussion

This survey was conducted to understand the perceptions of ophthalmologists, radiologists/radiation oncologists, and dermatologists about AI. These groups were selected as image analysis is a core work task for each profession and a variety of AI tools are being developed specifically for these specialties. To our knowledge, this survey is one of the first of its kind to investigate these specialist groups in parallel.

Most survey respondents perceived the introduction of AI technology in their respective fields as a positive advance. A recent survey of fellows and trainees of the Canadian Royal College of Physicians and Surgeons had similar findings: 72.2% of 3,919 respondents indicated that AI would have a positive impact on workflow and/or clinical practice and patient experience. Only 17.2% of respondents in the Canadian survey indicated that AI would have either a negative impact on workflow or place their specialty at risk. These generally positive sentiments regarding AI have been echoed across a range of medical specialties in previous surveys^13–16,19,22,23^, however, surveys of general practitioners^21^ and of psychiatrists^20^ have indicated that the potential of AI may be limited for these groups. Similar to our findings, low rates of clinical AI use by radiologists/radiation oncologists have previously been reported, indicating that AI has not yet been widely adopted in this field^15,16^. However, it is possible that AI use is underreported in surveys such as this due to a lack of visibility of algorithms that are deployed within imaging platforms or variations in the interpretation of what constitutes AI.

Most respondents considered that AI will have a noticeable impact on clinical practice within the next 5 years and on workforce needs within the next decade. In contrast, participants with more years of clinical experience were less likely to believe AI would have an impact on workforce needs within and beyond the coming decade, and current AI users were more likely to believe that workforce needs would increase. The current survey did not explore the basis for these diverging opinions. A survey of radiologists in the United States reported that AI would dramatically influence professional duties, however, the impact on workforce numbers was not ascertained^15^. Other surveys have indicated that workforce needs would remain stable or increase over the coming decade despite the introduction of AI^16,18^. Understanding the basis for these different perceptions on the impact of AI on the future medical workforce may be an interesting subject for further study.

Improved access to disease screening was reported by ophthalmologists and dermatologists as the greatest perceived advantage to the use of AI. Recognition of the need for increased disease screening capacity may be explained by the combined effects of workforce maldistribution in regional and rural areas versus urban areas and projected increases in the burden of diseases, such as diabetic retinopathy and skin cancer, due to population ageing and growth^29,30^. The perceived advantage of reducing time on monotonous tasks by radiologists/radiation oncologists is not surprising given the large and growing volume of images viewed by these practitioners. Other studies have similarly identified reduced administrative burden and decreased image interpretation time as advantages of AI use for primary care physicians^21^ and pathologists^16^.

The adoption of AI tools by clinicians is likely to be influenced by the extent to which they can be integrated into clinical workflows, enhance efficiency and achieve acceptable levels of performance^31^. The standards set by regulators of AI applications for health may not be directly aligned with the expectations of clinicians in practice. Interestingly, dermatologists were less accepting of a proposed clinical workflow which included AI-assisted diagnosis. This may be attributable to the distinct clinical practices of dermatologists^31^. Survey respondents had universally high expectations of AI system performance. There are many examples of AI outperforming humans^1,2^, however, until this study, little was known of the level of performance clinicians expect from AI systems.

In keeping with high expectations for AI system performance, respondents were concerned about medical liability due to machine error. Legal processes for dealing with harms arising from new technologies have evolved in parallel with innovation, however the legal, moral and ethical considerations are increasingly challenging as technologies become more autonomous^32,33^. Lessons learned from autonomous vehicle technologies may provide some guidance, but this remains an area for further research and broad-based consultation^33,34^.

An additional concern of respondents was a reduced reliance on medical specialists as a consequence of AI adoption. This concern is consistent with the impact of the technology on future workforce needs reported in this and other studies^15,16,19^. It is interesting that the primary concern of respondents was the divestment of healthcare to large technology companies. General mistrust in large technology companies has been documented recently^35^ and specifically in relation to healthcare^36^. Accordingly, numerous survey respondents called for leadership from specialist colleges and representative bodies to educate their members and to contribute to frameworks for the development, adoption and regulation of AI technologies in healthcare. The recent survey of fellows and residents conducted as part of the Canadian Royal College of Physicians and Surgeons Taskforce Report on Artificial Intelligence and Emerging Digital Technologies, similarly indicated that digital health literacy standards should be introduced and that governing bodies should take a proactive approach to AI^23^. Respondents called for improved training and education to improve awareness of AI and to increase proficiency in data science and statistics. Needs for training in ethics and legal aspects of AI ranked second to basic AI proficiency for fellows^23^.

The limitations of this survey warrant consideration. Volunteer response bias means that the results may not be broadly representative of the views of clinicians in Australia and New Zealand. Moreover, the survey may not be generalizable beyond these countries. As response rates from radiologists/radiation oncologists and dermatologists were low, it is not possible to ascertain whether the views of respondents are representative of others in these specialty groups. Finally, the survey design imposes limitations on the scope of response options and thus the survey findings should not be regarded as a comprehensive account of the perceptions of respondents. This limitation was mitigated by the inclusion of open-ended questions.

In conclusion, this survey highlights major similarities between the perceptions of ophthalmologists, radiologists/radiation oncologists and dermatologists in Australia and New Zealand about the application of AI in medicine. Overall, AI was regarded as a means to improve patient access to care, as well as to enhance clinical efficiency and performance. Concerns were raised about the influence of large technology and data companies, implications for medical liability and reduced reliance on medical specialists. As the successful implementation of AI in healthcare is dependent on detailed understanding of clinician and patient expectations of the technology, further research in these domains is needed.

## Methods

This prospective anonymous online survey was approved by the Human Research Ethics Committee of the Royal Victorian Eye and Ear Hospital (HREC 18-1408HL) and was conducted in accordance with the doctrines of the Declaration of Helsinki and its subsequent revisions. Electronic informed consent was obtained from each participant online prior to survey commencement.

### Survey design

Study data were collected and managed using REDCap electronic data capture tools hosted at the Centre for Eye Research Australia. Survey questions were developed after a review of previous survey literature and in consultation with ophthalmologists, radiologists and dermatologists to ensure face validity. Example scenarios and related occupational fields differed between surveys designed for each specialty. Draft surveys were circulated to executives from the RANZCO, the RANZCR and the ACD for testing. The survey consisted of 18 multiple choice and open-ended questions (Supplementary Table 1). An additional question was included for radiologists and radiation oncologists to enable differentiation between these professional groups. Questions focused on frequency of AI use, knowledge, workforce impact, preparedness for future AI implementation, acceptable levels of error, individual concerns and barriers. Survey invitations were emailed by college staff to all fellows and trainees of the colleges residing in Australia and New Zealand. Dermatology respondents were from Australia alone. Participation in this anonymous survey was voluntary and no incentives were provided.

### Data analysis

Multiple choice questions were analysed using Stata (v15.1, StataCorp, College Station, Texas). A complete-case analysis was conducted (i.e., incomplete surveys were excluded from the analyses, see Supplementary Table 3). Responses were compared according to profession, location of practice and years of experience using the Pearson’s *χ*^2^ test. Two-sided significance testing was conducted with an alpha of 5%. Thematic analysis of the response to “What do you think the college should do in preparation for the deployment of AI?” was undertaken using a ‘bottom up’ approach. Two authors (PR and XH) generated a list of initial codes after reviewing responses. Codes were built into broader categories and recurring themes were developed. Thematic discrepancies were resolved via discussion. Responses to questions regarding advantages and disadvantages of AI were analysed as follows: the first preference of each respondent received a score of 3, the second preference a score of 2 and the third preference a score of 1. Scores were tallied for each response and divided by the number of respondents to provide an overall score for each response. Higher scores indicate higher rankings. Scores were displayed as radar plots (Figs. 7, 8).

## Supplementary Information

Supplementary Tables.

## Data Availability

The authors declare that the data supporting the findings of this study are available within the paper and the supplementary information files. Raw data are available upon request.
