# Supplementary material for: A survey of clinicians on the use of artificial intelligence in ophthalmology, dermatology, radiology and radiation oncology
Source: Sci Rep. 2021 Mar 4;11:5193. doi: 10.1038/s41598-021-84698-5 (PMC7933437; doi:10.1038/s41598-021-84698-5)
Supplement: Supplementary file 1 — Supplementary Tables. [file 41598_2021_84698_MOESM1_ESM.docx]

**Supplementary Tables**

**Title:** A survey of clinicians on the use of artificial intelligence in ophthalmology, dermatology, radiology and radiation oncology

Authors:

^+^ Dr Jane Scheetz^1^

^+^ Dr Philip Rothschild^1,2^

Dr Myra McGuinness^1,3^

Dr Xavier Hadoux^1^

Professor H. Peter Soyer^4,5^

Professor Monika Janda^6^

Dr James Condon^7,8^

Dr Luke Oakden-Rayner^7,8^

Professor Lyle J. Palmer^7,8^

Dr Stuart Keel ^1^

* Associate Professor Peter van Wijngaarden^1,2^ (Corresponding author)

*^+^ These authors contributed equally to this work*

| **Supplementary Table 1 - Survey Questions** |  |
| --- | --- |
| 1. What is your specialty? *    - Radiology    - Radiation Oncology 2. Years of practice as an [insert specialist]:    - Currently in training    - < 5 years    - 5-10 years    - 10-20 years    - 20-30 years    - >30 years 3. Location of practice:    - Metropolitan    - Rural    - Both 4. How often do you use artificial intelligence software for your work as an [insert specialist]?    - Never    - Monthly    - Weekly    - Daily 5. What applications do you use artificial intelligence for?    - [Text box provided for respondents to type answer] 6. Relative to your colleagues in [insert specialty], how would you rate your knowledge of artificial intelligence and its application in the field of [insert specialty]?    - Excellent    - Above average    - Average    - Below average    - Very poor 7. How long do you think it will be before artificial intelligence has a noticeable impact on the field of [insert specialty]?    - Within 1 year    - 1-5 years    - 5-10 years    - >10 years    - Never 8. To what extent will artificial intelligence have an impact on [insert specialty] workforce needs within the next decade?    - To a great extent    - Somewhat    - Very little    - Not at all 9. To what extent will artificial intelligence have an impact on [insert specialty] workforce needs beyond the next decade?    - To a great extent    - Somewhat    - Very little    - Not at all 10. In what direction will the [insert specialty] workforce needs change due to artificial intelligence?     - Increase     - Decrease     - No change 11. Do you think that the [insert College] is adequately equipped to deal with the introduction of artificial intelligence in the field of [insert specialty]?     - Yes     - No     - Unsure | 1. What do you think that [insert college] should do in preparation for the deployment of artificial intelligence in [insert specialty]?    - [Text box provided for respondents to type answer] 2. What level of error is acceptable for artificial intelligence systems that are used for the purpose of screening for diseases, such as [diabetic retinopathy, mammography, or melanoma], by non-specialist healthcare workers?    - Equivalent to the worst performing [insert clinician]    - Equivalent to the average performing [insert clinician]    - Superior to the average performing [insert clinician]    - Equivalent to the best performing [insert clinician]    - Superior to the best performing [insert clinician] 3. What level of error is acceptable for artificial intelligence systems that are used for the purpose of diagnostic decision-support for [specialists]?    - Equivalent to the worst performing [insert clinician]    - Equivalent to the average performing [insert clinician]    - Superior to the average performing [insert clinician]    - Equivalent to the best performing [insert clinician]    - Superior to the best performing [insert clinician] 4. Would you consider using the following clinical workflow? Patients clinical images undergo artificial intelligence analysis. A [specialist] subsequently reviews both the image and the artificial intelligence findings.    - Yes    - No    - Unsure 5. Which of the following do you perceive as the greatest potential advantage of the use artificial intelligence systems in [insert specialty]? (rank the top 3 preferences where 1=greatest advantage)    - Improved patient access to disease screening    - More targeted referrals to specialist medical care    - More cost-effective health care    - Improved diagnostic confidence    - Reduced time spent by specialists on monotonous tasks    - Greater uniformity in diagnosis and management decisions    - More personalised and evidence-based disease management    - Improved prediction of disease outcomes    - Other 6. Which of the following do you perceive as the concern to the utilisation of artificial intelligence systems in [insert specialty]? (rank the top 3 preferences where 1=greatest drawback)    - Concerns over the divestment of health care to large technology and data companies    - Data security & privacy concerns    - Concerns over medical liability due to machine error    - Lack of confidence or trust in 'black-box' diagnosis    - Decreasing reliance on medical specialists for diagnosis and treatment advice    - Challenge to the fiduciary relationship between patient and doctor    - Concerns over benchmarking clinicians against machines    - Impact on workforce needs    - Other 7. Which professional group do you think will be most impacted by the introduction of AI in [insert specialty]?    1. For the RANZCO survey, available responses included: General practitioners, Optometrists, Ophthalmologists, Other.    2. For the RANZCR survey, available responses included: General practitioners, Radiographers and Sonographers, Radiologists / Radiation oncologists, Other.    3. For the ACD survey, available responses included: General Practitioners, Dermatologists, Other. 8. To what extent do you agree with the following statement: "the field of [insert specialty] will improve with the introduction of artificial intelligence"?    - Strongly agree    - Agree    - Neither agree nor disagree    - Disagree    - Strongly disagree   *This question was only included in the RANZCR survey, with possible options being ‘Radiology’ and ‘Radiation Oncology.’ |

| **Supplementary Table 2: Participant characteristics** | | | | | |
| --- | --- | --- | --- | --- | --- |
|  | **Specialty** | | |  |  |
|  | **RANZCO**  **(n=305)** | **RANZCR**  **(n=230)*** | **ACD**  **(n=97)** | **Total**  **(n=632)** | **Chi-squared statistic^‡^**  **p-value** |
| Location of practice |  |  |  |  |  |
| *Metro* | 206 (67.5%) | 180 (78.3%) | 74 (76.3%) | 460 (72.8%) | [χ](https://en.wikipedia.org/wiki/Chi_(letter))^2^ (4) = 8.89  p = 0.064 |
| *Rural* | 42 (13.8%) | 18 (7.8%) | 10 (10.3%) | 70 (11.1%) |  |
| *Both* | 57 (18.7%) | 32 (13.9%) | 13 (13.4%) | 102 (16.1%) |  |
| Years of experience |  |  |  |  |  |
| *Currently in training* | 30 (9.8%) | 26 (11.3%) | 10 (10.3%) | 66 (10.5%) | [χ](https://en.wikipedia.org/wiki/Chi_(letter))^2^ (10) = 10.21  p = 0.608 |
| *<5 years* | 34 (11.2%) | 18 (7.8%) | 10 (10.3%) | 62 (9.8%) |  |
| *5-10 years* | 28 (9.2%) | 25 (10.9%) | 17 (17.5%) | 70 (11.1%) |  |
| *10-20 years* | 60 (19.7%) | 52 (22.6%) | 19 (19.7%) | 131 (20.7%) |  |
| *20-30 years* | 80 (26.2%) | 54 (23.5%) | 21 (21.6%) | 155 (24.5%) |  |
| *>30 years* | 73 (23.9%) | 55 (23.9%) | 20 (20.6%) | 148 (23.4%) |  |
| *RANZCR participants were classified as either radiologists (n = 199) or radiation oncologists (n = 31) **^‡^** Pearson’s chi-squared test of independence with degrees of freedom. | | | | | |

| **Supplementary Table 3 - Partially completed survey responses** | | | | | |
| --- | --- | --- | --- | --- | --- |
|  | **College** | | |  |  |
|  | **RANZCO**  **(n=51)** | **RANZCR***  **(n=18)** | **ACD**  **(n=16)** | **Total**  **(n=85)** | **Chi-squared statistic^‡^**  **p-value** |
| Location |  |  |  |  |  |
| *Metro* | 31 (60.8%) | 13 (72.2%) | 13 (81.3%) | 57 (67.1%) | [χ](https://en.wikipedia.org/wiki/Chi_(letter))^2^ (4) = 4.53  p = 0.339 |
| *Rural* | 9 (17.6%) | 4 (22.2%) | 1 (6.3%) | 14 (16.5%) |  |
| *Both* | 11 (21.6%) | 1 (5.6%) | 2 (12.5%) | 14 (16.5%) |  |
| Years of practice |  |  |  |  |  |
| *Currently in training* | 7 (13.7%) | 3 (16.7%) | 2 (12.5%) | 12 (14.1%) | [χ](https://en.wikipedia.org/wiki/Chi_(letter))^2^ (10) = 9.80  p = 0.458 |
| *<5 years* | 5 (9.8%) | 0 (0%) | 1 (6.3%) | 6 (7.1%) |  |
| *5-10 years* | 7 (13.7%) | 4 (22.2%) | 3 (18.8%) | 14 (16.5%) |  |
| *10-20 years* | 5 (9.8%) | 6 (33.3%) | 4 (25.0%) | 15 (17.6%) |  |
| *20-30 years* | 10 (19.6%) | 2 (11.1%) | 3 (18.8%) | 15 (17.6%) |  |
| *>30 years* | 17 (33.3%) | 3 (16.7%) | 3 (18.8%) | 23 (27.1%) |  |
| *RANZCR participants were classified as either radiologists (n = 17) or radiation oncologists (n = 1) **^‡^** Pearson’s chi-squared test of independence with degrees of freedom. | | | | | |

| **Supplementary Table 4: Frequency and distribution of responses for speciality groups** | | | | | |
| --- | --- | --- | --- | --- | --- |
| **Question** | **Level** | **RANZCO** | **RANZCR** | **ACD** | **Chi-squared statistic***  **p-value** |
| **n** |  | 305 | 230 | 97 |  |
| **How often do you use artificial intelligence software for your work as an [insert specialist]?** | Never | 238 (78.0%) | 190 (82.6%) | 83 (85.6%) | [χ](https://en.wikipedia.org/wiki/Chi_(letter))^2^ (6) = 22.23  p = 0.001 |
|  | Monthly | 7 (2.3%) | 7 (3.0%) | 5 (5.2%) |  |
|  | Weekly | 12 (3.9%) | 19 (8.3%) | 4 (4.1%) |  |
|  | Daily | 48 (15.7%) | 14 (6.1%) | 5 (5.2%) |  |
| **Relative to your colleagues in [insert specialty], how would you rate your knowledge of artificial intelligence and its application in the field of [insert specialty]?** | Very Poor | 9 (3.0%) | 16 (7.0%) | 6 (6.2%) | [χ](https://en.wikipedia.org/wiki/Chi_(letter))^2^ (8) = 6.95  p = 0.542 |
|  | Below Average | 67 (22.0%) | 56 (24.3%) | 21 (21.6%) |  |
|  | Average | 148 (48.5%) | 107 (46.5%) | 46 (47.4%) |  |
|  | Above Average | 64 (21.0%) | 40 (17.4%) | 17 (17.5%) |  |
|  | Excellent | 17 (5.6%) | 11 (4.8%) | 7 (7.2%) |  |
| **How long do you think it will be before artificial intelligence has a noticeable impact on the field of [insert specialty]?** | Never | 0 (0%) | 0 (0%) | 0 (0%) | [χ](https://en.wikipedia.org/wiki/Chi_(letter))^2^ (6) = 15.72  p = 0.015 |
|  | More than 10 years | 15 (4.9%) | 21 (9.1%) | 10 (10.3%) |  |
|  | 5-10 years | 89 (29.2%) | 76 (33.0%) | 42 (43.3%) |  |
|  | 1-5 years | 176 (57.7%) | 122 (53.0%) | 41 (42.3%) |  |
|  | Within 1 year | 25 (8.2%) | 11 (4.8%) | 4 (4.1%) |  |
| **To what extent will artificial intelligence have an impact on [insert specialty] workforce needs within the next decade?** | Not at All | 12 (3.9%) | 5 (2.2%) | 1 (1.0%) | [χ](https://en.wikipedia.org/wiki/Chi_(letter))^2^ (6) = 13.38  p = 0.037 |
|  | Very Little | 82 (26.9%) | 50 (21.7%) | 30 (30.9%) |  |
|  | Somewhat | 151 (49.5%) | 106 (46.1%) | 49 (50.5%) |  |
|  | To a Great Extent | 60 (19.7%) | 69 (30.0%) | 17 (17.5%) |  |
| **To what extent will artificial intelligence have an impact on [insert specialty] workforce needs beyond the next decade?** | Not at All | 4 (1.3%) | 2 (0.9%) | 1 (1.0%) | [χ](https://en.wikipedia.org/wiki/Chi_(letter))^2^ (6) = 9.28  p = 0.158 |
|  | Very Little | 45 (14.8%) | 24 (10.4%) | 14 (14.4%) |  |
|  | Somewhat | 129 (42.3%) | 79 (34.3%) | 40 (41.2%) |  |
|  | To a Great Extent | 127 (41.6%) | 125 (54.3%) | 42 (43.3%) |  |
| **In what direction will the [insert specialty] workforce needs change due to artificial intelligence?** | Decrease | 100 (32.8%) | 71 (30.9%) | 29 (29.9%) | [χ](https://en.wikipedia.org/wiki/Chi_(letter))^2^ (4) = 1.09  p = 0.897 |
|  | No Change | 136 (44.6%) | 100 (43.5%) | 42 (43.3%) |  |
|  | Increase | 69 (22.6%) | 59 (25.7%) | 26 (26.8%) |  |
| **Do you think that the [insert College] is adequately equipped to deal with the introduction of artificial intelligence in the field of [insert specialty]?** | No | 91 (29.8%) | 89 (38.7%) | 36 (37.1%) | [χ](https://en.wikipedia.org/wiki/Chi_(letter))^2^ (4) = 5.89  p = 0.207 |
|  | Yes | 43 (14.1%) | 33 (14.3%) | 11 (11.3%) |  |
|  | Unsure | 171 (56.1%) | 108 (47.0%) | 50 (51.5%) |  |
| **What level of error is acceptable for artificial intelligence systems that are used for the purpose of screening for diseases, such as [diabetic retinopathy, mammography, or melanoma], by non-specialist healthcare workers?** | Equivalent to the worst performing specialist | 24 (7.9%) | 7 (3.0%) | 5 (5.2%) | [χ](https://en.wikipedia.org/wiki/Chi_(letter))^2^ (8) = 22.11  p = 0.005 |
|  | Equivalent to the average performing specialist | 105 (34.4%) | 57 (24.8%) | 29 (29.9%) |  |
|  | Superior to the average performing specialist | 71 (23.3%) | 57 (24.8%) | 19 (19.6%) |  |
|  | Equivalent to the best performing specialist | 73 (23.9%) | 59 (25.7%) | 24 (24.7%) |  |
|  | Superior to the best performing specialist | 32 (10.5%) | 50 (21.7%) | 20 (20.6%) |  |
| **What level of error is acceptable for artificial intelligence systems that are used for the purpose of diagnostic decision-support for [specialists]?** | Equivalent to the worst performing specialist | 7 (2.3%) | 11 (4.8%) | 3 (3.1%) | [χ](https://en.wikipedia.org/wiki/Chi_(letter))^2^ (8) = 16.57  p = 0.035 |
|  | Equivalent to the average performing specialist | 54 (17.7%) | 40 (17.4%) | 11 (11.3%) |  |
|  | Superior to the average performing specialist | 58 (19.0%) | 55 (23.9%) | 22 (22.7%) |  |
|  | Equivalent to the best performing specialist | 128 (42.0%) | 69 (30.0%) | 31 (32.0%) |  |
|  | Superior to the best performing specialist | 58 (19.0%) | 55 (23.9%) | 30 (30.9%) |  |
| **Would you consider using the following clinical workflow? Patients clinical images undergo artificial intelligence analysis. A [specialist] subsequently reviews both the image and the artificial intelligence findings.** | No | 37 (12.1%) | 10 (4.3%) | 12 (12.4%) | [χ](https://en.wikipedia.org/wiki/Chi_(letter))^2^ (4) = 28.60  p = <0.001 |
|  | Yes | 250 (82.0%) | 190 (82.6%) | 65 (67.0%) |  |
|  | Unsure | 18 (5.9%) | 30 (13.0%) | 20 (20.6%) |  |
| **To what extent do you agree with the following statement: "the field of [insert specialty] will improve with the introduction of artificial intelligence"?** | Strongly Disagree | 5 (1.6%) | 6 (2.6%) | 1 (1.0%) | [χ](https://en.wikipedia.org/wiki/Chi_(letter))^2^ (8) = 9.37  p = 0.312 |
|  | Disagree | 12 (3.9%) | 7 (3.0%) | 5 (5.2%) |  |
|  | Neither agree nor disagree | 64 (21.0%) | 57 (24.8%) | 26 (26.8%) |  |
|  | Agree | 145 (47.5%) | 116 (50.4%) | 51 (52.6%) |  |
|  | Strongly Agree | 79 (25.9%) | 44 (19.1%) | 14 (14.4%) |  |
| * Pearson’s chi-squared test of independence with degrees of freedom. | | | | | |

| **Supplementary Table 5 – Types of AI Applications Used** | | | |
| --- | --- | --- | --- |
| Ophthalmology (67 respondents) | | | |
| How is AI used in ophthalmology? | n | How is AI used in ophthalmology? | n |
| Glaucoma assessment / Humphrey visual fields / progression analysis | 26 | Autorefraction | 2 |
| Optical Coherence Tomography | 25 | MRI protocols | 1 |
| Retinal diagnosis and analysis | 8 | Stealthy navigation in orbital surgery | 1 |
| Lens power calculations (IOLmaster) | 7 | Image acquisition optimisation | 1 |
| Keratometry | 3 | Axial length | 1 |
| Image analysis | 3 | Fluorescein angiography | 1 |
| Grading diabetic retinopathy | 3 |  |  |
| Radiology/Radiation Oncology (40 respondents) | | | |
| How is AI used in radiology / radiation oncology? | n | How is AI used in radiology / radiation oncology? | n |
| Computer aided detection (CAD) for lesions on CT and MRI (including brain, spine, chest, coronary arteries, breast, prostate, and colon) | 12 | Bone age determination | 2 |
| CT Colonography (General Electric) software | 10 | RAPID CT Perfusion for stroke investigations | 2 |
| CT scanning (Blackford and Zebra apps, hanging protocol, window preset, image reconstruction) | 5 | Mammogram image enhancement | 1 |
| Lung nodule assessment | 5 | Cardiovascular measurements | 1 |
| (Auto)contouring and planning radiotherapy (including with Raysearch) | 5 | Brain morphometric analysis | 1 |
| Assess breast density | 2 | Neurostat analysis of Brain SPECT and PET | 1 |
| Dermatology (8 respondents) | | | |
| How is AI used in dermatology? | n | How is AI used in dermatology? | n |
| Mole mapping (E.g. Fotofinder) & analysis | 4 | Image analysis software | 2 |
| Vectra 3D skin photography | 2 |  |  |
|  | | | |
| General AI Use (43 respondents; specialties aggregated ) | | | |
| How is AI used in general? | n | How is AI used in general? | n |
| Google / search functions | 17 | Predictive text messages | 2 |
| Dictation software | 6 | Apps | 2 |
| Clinical audit functions | 5 | Computer security | 1 |
| Email | 4 | Maps | 1 |
| Data mining | 2 | Profitability analysis reports | 1 |
| Calendars | 2 |  |  |

| **Supplementary Table 6: Five direct quotes for each of the top five themes emerging from the qualitative responses** | |
| --- | --- |
| **Theme 1:** | **Theme 2** |
| **A need for improved training and education of college members about AI** | **The need for the colleges to be proactive in their approach in order to safeguard member interests** |
| **Quotes** | **Quotes** |
| “Online education for current fellows and trainees. Understanding of the implementation, and practical implications of AI.” | “Partner with industry and academia to prioritise, shape and assess AI platforms. If [the College] isn't involved then others will take the lead.” |
| “Clearly explain to fellows the issues that exist - I think this will be a challenge to work out how we can safely use these technologies in the context of global tech giants / confidentiality etc.” | “Actively assess potential uses and hence the likely effect on [clinical] practice. Based on this determine the directions the college should be taking in the introduction of these technologies” |
| “Collaborate with technological companies and introduce courses/lectures at [the College] meeting” | “Be at the forefront of policy and licensing.” |
| “Conduct regular sessions to guide the [clinician] with latest development. May need an expert team to conduct those changes effectively” | “Be intimately involved in the trial and application of emerging AI systems, work closely with government and other colleges in having a unified approach to implementation policies” |
| “Provide data to the membership of changes in all the [clinical] sector so we are informed, can inform our patients and be prepared for the change to practice.” | “Continue monitoring the area generally and its application in business and medicine.” |
| **Theme 3** | **Theme 4** |
| **The development of frameworks and/or guidelines for AI implementation by the colleges, or working groups** | **Concerns about the impact on workforce needs** |
| **Quotes** | **Quotes** |
| “Appoint a committee, setup a leadership team to explore the implication, potential and regulatory framework within this growing field. Select those who are actively working in this field, those with hands-on experience with AI.” | “Ensure adequate jobs for current and training [clinicians]” |
| “Provide an ethical framework for [clinicians] to address AI in the workplace. For example, [clinicians] will still ultimately be responsible for any AI platforms used in their workplaces and responsibility will still lie with the treating doctor for decision making.” | “Do not train too many new registrars - we are at risk of producing an over-supply of [clinicians].” |
| “Set ethical framework for data access and standard for data labelling quality assessment. Medico-legal guidance - who is to blame if error has been made based on information provided by AI.” | “Look at ways [clinicians] can be incorporated into AI screening and diagnosis rather than AI stand alone” |
| “We need a collaborative task force to be at the forefront of this unavoidable future. AI should be adapted to be complementary to practice. If the [clinical] AI introduced into medicine is not developed with the aid of [clinicians] then there will be poor outcomes for patients and practitioners. We need to be leaders in this.” | “Start preparing the government to allow fewer registrars to be trained in order to prevent workforce oversupply” |
| “Form a subcommittee which is tasked to write a position paper for [the College] on AI in [the specialty] (a) now, (b) next few years, (c) beyond and options how we should respond to these challenges.” | “maintaining the lead in conversation, keeping [the clinician] using the technology rather than being replaced by it” |
| **Theme 5** |  |
| **The development of measures to ensure patient safety** |  |
| **Quotes** |  |
| “Ensure rigorous safety is in place before AI is considered mainstream” |  |
| “Make sure any changes are in the best interests of patients and our profession” |  |
| “Engage at the government level to ensure safeguards are in place for regulating AI use, so that patient care is not compromised” |  |
| “Embrace technology that improves clinical outcomes” |  |
| “All applications will need to be validated and all abnormal results will need to be confirmed by a [clinician] (See Boeing Artificial Intelligence driving two Max 8 jets into the ground).” |  |

| **Supplementary Table 7 - Sub-group analyses based on location** | | | | | |
| --- | --- | --- | --- | --- | --- |
| **Question** | **Level** | **Metropolitan** | **Rural** | **Both** | **Chi-squared statistic***  **p-value** |
| **n** |  | **460** | **70** | **102** |  |
| **How often do you use artificial intelligence software for your work as an [insert specialist]?** | Never | 373 (81.1%) | 59 (84.3%) | 79 (77.5%) | [χ](https://en.wikipedia.org/wiki/Chi_(letter))^2^ (6) = 3.49  p = 0.745 |
|  | Monthly | 16 (3.5%) | 1 (1.4%) | 2 (2.0%) |  |
|  | Weekly | 23 (5.0%) | 4 (5.7%) | 8 (7.8%) |  |
|  | Daily | 48 (10.4%) | 6 (8.6%) | 13 (12.7%) |  |
| **Relative to your colleagues in [insert specialty], how would you rate your knowledge of artificial intelligence and its application in the field of [insert specialty]?** | Very Poor | 24 (5.2%) | 3 (4.3%) | 4 (3.9%) | [χ](https://en.wikipedia.org/wiki/Chi_(letter))^2^ (8) = 8.45  p = 0.391 |
|  | Below Average | 101 (22.0%) | 19 (27.1%) | 24 (23.5%) |  |
|  | Average | 214 (46.5%) | 40 (57.1%) | 47 (46.1%) |  |
|  | Above Average | 93 (20.2%) | 7 (10.0%) | 21 (20.6%) |  |
|  | Excellent | 28 (6.1%) | 1 (1.4%) | 6 (5.9%) |  |
| **How long do you think it will be before artificial intelligence has a noticeable impact on the field of [insert specialty]?** | More than 10 years | 37 (8.0%) | 4 (5.7%) | 5 (4.9%) | [χ](https://en.wikipedia.org/wiki/Chi_(letter))^2^ (6) = 3.50  p = 0.743 |
|  | 5-10 years | 148 (32.2%) | 23 (32.9%) | 36 (35.3%) |  |
|  | 1-5 years | 244 (53.0%) | 41 (58.6%) | 54 (52.9%) |  |
|  | Within 1 year | 31 (6.7%) | 2 (2.9%) | 7 (6.9%) |  |
| **To what extent will artificial intelligence have an impact on [insert specialty] workforce needs within the next decade?** | Not at All | 14 (3.0%) | 1 (1.4%) | 3 (2.9%) | [χ](https://en.wikipedia.org/wiki/Chi_(letter))^2^ (6) = 3.47  p = 0.747 |
|  | Very Little | 116 (25.2%) | 21 (30.0%) | 25 (24.5%) |  |
|  | Somewhat | 221 (48.0%) | 37 (52.9%) | 48 (47.1%) |  |
|  | To a Great Extent | 109 (23.7%) | 11 (15.7%) | 26 (25.5%) |  |
| **To what extent will artificial intelligence have an impact on [insert specialty] workforce needs beyond the next decade?** | Not at All | 7 (1.5%) | 0 (0.0%) | 0 (0.0%) | [χ](https://en.wikipedia.org/wiki/Chi_(letter))^2^ (6) = 6.48  p = 0.371 |
|  | Very Little | 54 (11.7%) | 14 (20.0%) | 15 (14.7%) |  |
|  | Somewhat | 181 (39.3%) | 27 (38.6%) | 40 (39.2%) |  |
|  | To a Great Extent | 218 (47.4%) | 29 (41.4%) | 47 (46.1%) |  |
| **In what direction will the [insert specialty] workforce needs change due to artificial intelligence?** | Decrease | 135 (29.3%) | 22 (31.4%) | 43 (42.2%) | [χ](https://en.wikipedia.org/wiki/Chi_(letter))^2^ (4) = 6.76  p = 0.149 |
|  | No Change | 212 (46.1%) | 30 (42.9%) | 36 (35.3%) |  |
|  | Increase | 113 (24.6%) | 18 (25.7%) | 23 (22.5%) |  |
| **Do you think that the [insert College] is adequately equipped to deal with the introduction of artificial intelligence in the field of [insert specialty]?** | No | 157 (34.1%) | 20 (28.6%) | 39 (38.2%) | [χ](https://en.wikipedia.org/wiki/Chi_(letter))^2^ (4) = 3.60  p = 0.463 |
|  | Yes | 67 (14.6%) | 11 (15.7%) | 9 (8.8%) |  |
|  | Unsure | 236 (51.3%) | 39 (55.7%) | 54 (52.9%) |  |
| **What level of error is acceptable for artificial intelligence systems that are used for the purpose of screening for diseases, such as [diabetic retinopathy, mammography, or melanoma], by non-specialist healthcare workers?** | Equivalent to the worst performing specialist | 22 (4.8%) | 5 (7.1%) | 9 (8.8%) | [χ](https://en.wikipedia.org/wiki/Chi_(letter))^2^ (8) = 11.60  p = 0.170 |
|  | Equivalent to the average performing specialist | 141 (30.7%) | 20 (28.6%) | 30 (29.4%) |  |
|  | Superior to the average performing specialist | 104 (22.6%) | 22 (31.4%) | 21 (20.6%) |  |
|  | Equivalent to the best performing specialist | 121 (26.3%) | 16 (22.9%) | 19 (18.6%) |  |
|  | Superior to the best performing specialist | 72 (15.7%) | 7 (10.0%) | 23 (22.5%) |  |
| **What level of error is acceptable for artificial intelligence systems that are used for the purpose of diagnostic decision-support for [specialists]?** | Equivalent to the worst performing specialist | 16 (3.5%) | 3 (4.3%) | 2 (2.0%) | [χ](https://en.wikipedia.org/wiki/Chi_(letter))^2^ (8) = 8.22  p = 0.412 |
|  | Equivalent to the average performing specialist | 78 (17.0%) | 11 (15.7%) | 16 (15.7%) |  |
|  | Superior to the average performing specialist | 91 (19.8%) | 20 (28.6%) | 24 (23.5%) |  |
|  | Equivalent to the best performing specialist | 169 (36.7%) | 27 (38.6%) | 32 (31.4%) |  |
|  | Superior to the best performing specialist | 106 (23.0%) | 9 (12.9%) | 28 (27.5%) |  |
| **Would you consider using the following clinical workflow? Patients clinical images undergo artificial intelligence analysis. A [specialist] subsequently reviews both the image and the artificial intelligence findings.** | No | 44 (9.6%) | 9 (12.9%) | 6 (5.9%) | [χ](https://en.wikipedia.org/wiki/Chi_(letter))^2^ (4) = 5.39  p = 0.249 |
|  | Yes | 361 (78.5%) | 57 (81.4%) | 87 (85.3%) |  |
|  | Unsure | 55 (12.0%) | 4 (5.7%) | 9 (8.8%) |  |
| **To what extent do you agree with the following statement: "the field of [insert specialty] will improve with the introduction of artificial intelligence"?** | Strongly Disagree | 11 (2.4%) | 0 (0.0%) | 1 (1.0%) | [χ](https://en.wikipedia.org/wiki/Chi_(letter))^2^ (8) = 10.55  p = 0.229 |
|  | Disagree | 20 (4.3%) | 3 (4.3%) | 1 (1.0%) |  |
|  | Neither agree nor disagree | 103 (22.4%) | 18 (25.7%) | 26 (25.5%) |  |
|  | Agree | 228 (49.6%) | 39 (55.7%) | 45 (44.1%) |  |
|  | Strongly Agree | 98 (21.3%) | 10 (14.3%) | 29 (28.4%) |  |
| * Pearson’s chi-squared test of independence with degrees of freedom. | | | | | |

| **Supplementary Table 8 - Sub-group analyses based on years of experience** | | | | | | | | |
| --- | --- | --- | --- | --- | --- | --- | --- | --- |
| **Question** | **Level** | **Currently in training** | **Less than 5** | **5-10** | **10-20** | **20-30** | **>30** | **Chi-squared statistic***  **p-value** |
| **n** |  | **66** | **62** | **70** | **131** | **155** | **148** |  |
| **How often do you use artificial intelligence software for your work as an [insert specialist]?** | Never | 56 (84.8%) | 52 (83.9%) | 62 (88.6%) | 104 (79.4%) | 121 (78.1%) | 116 (78.4%) | [χ](https://en.wikipedia.org/wiki/Chi_(letter))^2^ (15) = 10.18,  p = 0.808 |
|  | Monthly | 1 (1.5%) | 3 (4.8%) | 2 (2.9%) | 3 (2.3%) | 5 (3.2%) | 5 (3.4%) |  |
|  | Weekly | 3 (4.5%) | 3 (4.8%) | 3 (4.3%) | 6 (4.6%) | 12 (7.7%) | 8 (5.4%) |  |
|  | Daily | 6 (9.1%) | 4 (6.5%) | 3 (4.3%) | 18 (13.7%) | 17 (11.0%) | 19 (12.8%) |  |
| **Relative to your colleagues in [insert specialty], how would you rate your knowledge of artificial intelligence and its application in the field of [insert specialty]?** | Very Poor | 3 (4.5%) | 3 (4.8%) | 0 (0.0%) | 6 (4.6%) | 9 (5.8%) | 10 (6.8%) | [χ](https://en.wikipedia.org/wiki/Chi_(letter))^2^ (20) = 25.55,  p = 0.181 |
|  | Below Average | 15 (22.7%) | 8 (12.9%) | 16 (22.9%) | 29 (22.1%) | 35 (22.6%) | 41 (27.7%) |  |
|  | Average | 32 (48.5%) | 35 (56.5%) | 31 (44.3%) | 62 (47.3%) | 73 (47.1%) | 68 (45.9%) |  |
|  | Above Average | 9 (13.6%) | 10 (16.1%) | 17 (24.3%) | 26 (19.8%) | 31 (20.0%) | 28 (18.9%) |  |
|  | Excellent | 7 (10.6%) | 6 (9.7%) | 6 (8.6%) | 8 (6.1%) | 7 (4.5%) | 1 (0.7%) |  |
| **How long do you think it will be before artificial intelligence has a noticeable impact on the field of [insert specialty]?** | More than 10 years | 6 (9.1%) | 2 (3.2%) | 3 (4.3%) | 7 (5.3%) | 17 (11.0%) | 11 (7.4%) | [χ](https://en.wikipedia.org/wiki/Chi_(letter))^2^ (15) = 23.47,  p = 0.075 |
|  | 5-10 years | 28 (42.4%) | 23 (37.1%) | 29 (41.4%) | 48 (36.6%) | 37 (23.9%) | 42 (28.4%) |  |
|  | 1-5 years | 29 (43.9%) | 30 (48.4%) | 37 (52.9%) | 67 (51.1%) | 91 (58.7%) | 85 (57.4%) |  |
|  | Within 1 year | 3 (4.5%) | 7 (11.3%) | 1 (1.4%) | 9 (6.9%) | 10 (6.5%) | 10 (6.8%) |  |
| **To what extent will artificial intelligence have an impact on [insert specialty] workforce needs within the next decade?** | Not at All | 1 (1.5%) | 0 (0.0%) | 0 (0.0%) | 3 (2.3%) | 7 (4.5%) | 7 (4.7%) | [χ](https://en.wikipedia.org/wiki/Chi_(letter))^2^ (15) = 45.01,  p = <0.001 |
|  | Very Little | 7 (10.6%) | 12 (19.4%) | 14 (20.0%) | 38 (29.0%) | 53 (34.2%) | 38 (25.7%) |  |
|  | Somewhat | 31 (47.0%) | 28 (45.2%) | 34 (48.6%) | 71 (54.2%) | 66 (42.6%) | 76 (51.4%) |  |
|  | To a Great Extent | 27 (40.9%) | 22 (35.5%) | 22 (31.4%) | 19 (14.5%) | 29 (18.7%) | 27 (18.2%) |  |
| **To what extent will artificial intelligence have an impact on [insert specialty] workforce needs beyond the next decade?** | Not at All | 0 (0.0%) | 0 (0.0%) | 0 (0.0%) | 1 (0.8%) | 4 (2.6%) | 2 (1.4%) | [χ](https://en.wikipedia.org/wiki/Chi_(letter))^2^ (15) = 42.95,  p = <0.001 |
|  | Very Little | 5 (7.6%) | 2 (3.2%) | 6 (8.6%) | 19 (14.5%) | 24 (15.5%) | 27 (18.2%) |  |
|  | Somewhat | 17 (25.8%) | 19 (30.6%) | 24 (34.3%) | 53 (40.5%) | 67 (43.2%) | 68 (45.9%) |  |
|  | To a Great Extent | 44 (66.7%) | 41 (66.1%) | 40 (57.1%) | 58 (44.3%) | 60 (38.7%) | 51 (34.5%) |  |
| **In what direction will the [insert specialty] workforce needs change due to artificial intelligence?** | Decrease | 21 (31.8%) | 27 (43.5%) | 28 (40.0%) | 45 (34.4%) | 52 (33.5%) | 27 (18.2%) | [χ](https://en.wikipedia.org/wiki/Chi_(letter))^2^ (10) = 24.42,  p = 0.007 |
|  | No Change | 30 (45.5%) | 22 (35.5%) | 23 (32.9%) | 56 (42.7%) | 74 (47.7%) | 73 (49.3%) |  |
|  | Increase | 15 (22.7%) | 13 (21.0%) | 19 (27.1%) | 30 (22.9%) | 29 (18.7%) | 48 (32.4%) |  |
| **Do you think that the [insert College] is adequately equipped to deal with the introduction of artificial intelligence in the field of [insert specialty]?** | No | 22 (33.3%) | 30 (48.4%) | 32 (45.7%) | 50 (38.2%) | 46 (29.7%) | 36 (24.3%) | [χ](https://en.wikipedia.org/wiki/Chi_(letter))^2^ (10) = 32.77,  p = <0.001 |
|  | Yes | 5 (7.6%) | 3 (4.8%) | 4 (5.7%) | 15 (11.5%) | 26 (16.8%) | 34 (23.0%) |  |
|  | Unsure | 39 (59.1%) | 29 (46.8%) | 34 (48.6%) | 66 (50.4%) | 83 (53.5%) | 78 (52.7%) |  |
| What level of error is acceptable for artificial intelligence systems that are used for the purpose of screening for diseases, such as [diabetic retinopathy, mammography, or melanoma], by non-specialist healthcare workers? | Equivalent to the worst performing specialist | 4 (6.1%) | 4 (6.5%) | 7 (10.0%) | 7 (5.3%) | 7 (4.5%) | 7 (4.7%) | [χ](https://en.wikipedia.org/wiki/Chi_(letter))^2^ (20) = 12.98,  p = 0.878 |
|  | Equivalent to the average performing specialist | 25 (37.9%) | 17 (27.4%) | 22 (31.4%) | 37 (28.2%) | 47 (30.3%) | 43 (29.1%) |  |
|  | Superior to the average performing specialist | 16 (24.2%) | 15 (24.2%) | 16 (22.9%) | 32 (24.4%) | 40 (25.8%) | 28 (18.9%) |  |
|  | Equivalent to the best performing specialist | 13 (19.7%) | 17 (27.4%) | 17 (24.3%) | 28 (21.4%) | 38 (24.5%) | 43 (29.1%) |  |
|  | Superior to the best performing specialist | 8 (12.1%) | 9 (14.5%) | 8 (11.4%) | 27 (20.6%) | 23 (14.8%) | 27 (18.2%) |  |
| What level of error is acceptable for artificial intelligence systems that are used for the purpose of diagnostic decision-support for [specialists]? | Equivalent to the worst performing specialist | 2 (3.0%) | 1 (1.6%) | 3 (4.3%) | 5 (3.8%) | 5 (3.2%) | 5 (3.4%) | [χ](https://en.wikipedia.org/wiki/Chi_(letter))^2^ (20) = 18.35,  p = 0.565 |
|  | Equivalent to the average performing specialist | 17 (25.8%) | 6 (9.7%) | 13 (18.6%) | 21 (16.0%) | 25 (16.1%) | 23 (15.5%) |  |
|  | Superior to the average performing specialist | 16 (24.2%) | 12 (19.4%) | 20 (28.6%) | 26 (19.8%) | 36 (23.2%) | 25 (16.9%) |  |
|  | Equivalent to the best performing specialist | 17 (25.8%) | 24 (38.7%) | 19 (27.1%) | 49 (37.4%) | 61 (39.4%) | 58 (39.2%) |  |
|  | Superior to the best performing specialist | 14 (21.2%) | 19 (30.6%) | 15 (21.4%) | 30 (22.9%) | 28 (18.1%) | 37 (25.0%) |  |
| Would you consider using the following clinical workflow? Patients clinical images undergo artificial intelligence analysis. A [specialist] subsequently reviews both the image and the artificial intelligence findings. | No | 5 (7.6%) | 5 (8.1%) | 9 (12.9%) | 11 (8.4%) | 13 (8.4%) | 16 (10.8%) | [χ](https://en.wikipedia.org/wiki/Chi_(letter))^2^ (10) = 10.00,  p = 0.441 |
|  | Yes | 50 (75.8%) | 53 (85.5%) | 54 (77.1%) | 106 (80.9%) | 131 (84.5%) | 111 (75.0%) |  |
|  | Unsure | 11 (16.7%) | 4 (6.5%) | 7 (10.0%) | 14 (10.7%) | 11 (7.1%) | 21 (14.2%) |  |
| To what extent do you agree with the following statement: "the field of [insert specialty] will improve with the introduction of artificial intelligence"? | Strongly Disagree | 1 (1.5%) | 1 (1.6%) | 1 (1.4%) | 0 (0.0%) | 8 (5.2%) | 1 (0.7%) | [χ](https://en.wikipedia.org/wiki/Chi_(letter))^2^ (20) = 29.01,  p = 0.088 |
|  | Disagree | 2 (3.0%) | 0 (0%) | 5 (7.1%) | 6 (4.6%) | 3 (1.9%) | 8 (5.4%) |  |
|  | Neither agree nor disagree | 18 (27.3%) | 14 (22.6%) | 10 (14.3%) | 33 (25.2%) | 41 (26.5%) | 31 (30.0%) |  |
|  | Agree | 27 (40.9%) | 36 (58.1%) | 37 (52.9%) | 67 (51.1%) | 73 (47.1%) | 72 (48.7%) |  |
|  | Strongly Agree | 18 (27.3%) | 11 (17.7%) | 17 (24.3%) | 25 (19.1%) | 30 (19.4%) | 36 (24.3%) |  |
| * Pearson’s chi-squared test of independence with degrees of freedom. | | | | | | | | |
